# Simulating the Voltage-Dependent Fluorescence of Di-8-ANEPPS in a Lipid Membrane

**DOI:** 10.1021/acs.jpclett.3c01257

**Published:** 2023-09-07

**Authors:** Rachael Youngworth, Benoît Roux

**Affiliations:** † Department of Chemistry, The University of Chicago, 5735 S. Ellis Avenue, Chicago, Illinois 60637, United States; ‡ Department of Biochemistry and Molecular Biology, The University of Chicago, 929 E. 57th Street W225, Chicago, Illinois 60637, United States

## Abstract

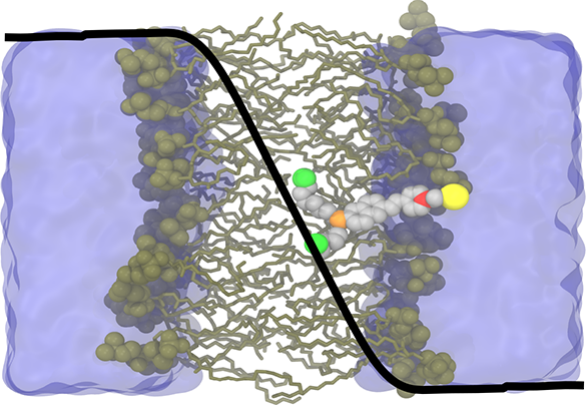

Voltage-sensitive fluorescent dyes such as di-8-ANEPPS (di-8-aminonaphthylethylenepyridinium propylsulfonate) are powerful tools to study biological membranes. Its fluorescence is affected by changes in the membrane potential and other factors, requiring extensive calibration to extract meaningful quantitative results. The amphiphilic di-8-ANEPPS molecule is expected to bind at the membrane-solution interface. However, atomic-level information is sparse about its position and orientation in the membrane, especially in regards to how the latter dynamically fluctuates to affect the observed fluorescence. In the present work, molecular dynamics simulations of the ground and excited states of di-8-ANEPPS embedded in a DPPC membrane as represented by classical force fields were used to investigate how the fluorescence is affected by externally applied potential. The calculations reproduce the shifts in the wavelength of emission as a function of voltage that are observed experimentally, indicating that the approach can help better understand the various factors that can affect the fluorescence of membrane-bound dyes.

Fluorescent dyes such as di-4-ANEPPS and di-8-ANEPPS are highly sensitive fluorescent dyes displaying consistent potentiometric responses in a wide variety of systems.^1−3^ While they have similar spectral properties, di-8-ANEPPS is less water-soluble and more stable in the membrane than di-4-ANEPPS due to its longer hydrophobic carbon tails.^1−3^ Fluorescence measurements with di-8-ANEPPS can be used to detect changes in the interfacial membrane dipole potential,^4−8^ to see how it is affected by multiple factors.^9−17^ It can also be used to directly probe the voltage regulation of ion channels,^18^ and even monitor neuronal activity.^19,20^ However, careful calibration of the potentiometric dye is required to extract meaningful quantitative results from the measurements because a number of complex factors contribute to the observed signal.

At the molecular level, both di-4-ANEPPS and di-8-ANEPPS are amphiphilic zwitterions. The hydrophilic negatively charged sulfonate headgroup is expected to reside near the membrane-solution interface while the nonpolar hydrocarbon tails are localized within the hydrophobic core of the bilayer. In the ground state, the pyridinium nitrogen, which is separated from the sulfonate group by a short propyl chain, carries a corresponding positive charge. In the excited state, the flow of charge along the conjugated structure of the pyridinium, ethylene, and naphthyl rings causes the amino nitrogen to be positively charged. Qualitatively, the increase in charge separation during the transition from the ground to the excited state results in a positive charge penetrating the membrane. This simple picture, however, must be reconciled with the tumultuous dynamic environment of a solvated membrane. For instance, whether there are significant differences in the average orientation of the dye when it is in its ground or excited state and how this might affect the potentiometric measurements is unclear.

Our goal is to gain atomic-level insight into the factors affecting the fluorescence of di-8-ANEPPS in response to an applied membrane potential using molecular dynamics (MD) simulations based on atomic models. Classical^21−23^ and QM/MM^24,25^ simulation studies of di-8-ANEPPS in a variety of complex environments have previously been carried out, but none that directly tackled the influence of the membrane potential. QM/MM approaches are attractive because they are based on fundamental principles. However, these sophisticated approaches are computationally demanding, increasing the difficulty in achieving an adequate configurational sampling of a solvated lipid membrane. On the other hand, classical MD simulations based on molecular mechanical force fields are certainly simpler and more approximate. But this simplicity makes it possible to carry out the μs-scale simulations that are necessary to accurately probe the effect of the membrane potential on the fluorescence of di-8-ANEPPS. Here, relying on the di-8-ANEPPS force fields introduced previously,^23^ the change in absorption and emission wavelengths of the fluorescent dye as a function of the membrane potential was calculated. The results show that the model is able to reproduce the experimentally observed shifts in the emission wavelength as a function of voltage, suggesting that MD simulations based on classical force fields may help us better understand the various factors affecting the fluorescence of voltage-sensitive dyes used in biological studies.

## Methods

The generation of the nonpolarizable force fields for di-8-ANEPPS in its ground and first excited state was detailed in a previous study modeling di-8-ANEPPS in a series of solvents.^23^ Details about the theoretical chemistry methodology are provided in Section 1 of the Supporting Information. Briefly, the optimization of the force field for the ground and excited states was carried out using the GAAMP (General Automated Atomic Model Parameterization)^26^ with initial parameters from the CHARMM General Force Field (CGenFF).^27^ Parameterization of the excited state involved ab initio calculations using the CASSCF method, which is characterized by the categorization of orbital configurations into core, active, or virtual (core orbitals are fully occupied, virtual orbitals are fully unoccupied, and the active orbitals are partially occupied because their occupancy changes over the course of a transition). Selection of the correct active orbitals is critical to the viability of the CASSCF method. Previous CASSCF studies provided essential information.^24,25^ Robinson et al. previously showed that the transition to the first excited state singlet of a reduced fragment of di-8-ANEPPS can be accurately represented with a CASSCF(6,6) calculation (where 6 electrons and 6 orbitals are considered to be the active space).^24^ This fragment is essentially the conjugated portion of the molecule, cutting off the two hydrocarbon tails and the negatively charged propyl sulfonate. The CASSCF calculation on this reduced molecule was repeated before the attempt of a CASSCF calculation with the di-4-ANEPPS molecule. For convenience, the shorter molecule di-4-ANEPPS was used to carry out the parametrization, and the parameters were extended to di-8-ANEPPS afterward. The QM electrostatic potential data used in the charge fitting protocol to generate force field parameters reflecting the excited state was generated using the ORCA program system (version 4.0).^28,29^ Convergence was achieved at the def2-SVP def2-SVP/C level with orbstep SuperCi and switchstep DIIS convergence criteria included. The calculation reflected the excited state after an instantaneous electronic transition from the ground state without any geometry optimization. The dipole of the model of the ground state of di-8-ANEPPS is 31.2 D, and the dipole of the excited state is 41.4 D, a global change of about 10.2 D. These electrostatic features of the optimized force field reproduce the QM calculations and are consistent with a previous QM study reporting a ground state dipole of 36.7 ± 2.7 D and an excited state dipole of 48.3 ± 2.6 D, for a total change of 11.4 ± 1.7 D.^25^ The parameters for the water and DPPC molecules were taken from the C36 CHARMM force field.^30^ All force fields for di-8-ANEPPS in its ground and LE excited states parameter files are given in https://github.com/RouxLab/Fluorescence-of-Di-8-ANEPPS.

A pure DPPC membrane system without di-8-ANEPPS was constructed and simulated for a minimum of 250 ns at a temperature of 323.15 K and constant volume with applied transmembrane potentials of −500 mV, −100 mV, 0 mV, 100 mV, and 500 mV. The system comprises 50 DPPC molecules in each leaflet and a total of 4143 water molecules. An additional set of membrane systems was constructed with di-8-ANEPPS inserted into one of the leaflets in an orientation parallel to the membrane normal and simulated with the same series of applied transmembrane potentials. Trajectories of 1 μs were generated with di-8-ANEPPS in the ground and excited states at a temperature of 323.15 K with a constant volume. All simulated systems were constructed using the CHARMM-GUI membrane builder module.^31−36^ To model membrane potential in molecular dynamics, a uniform, external electric field acting on all charged particles is applied perpendicular to the plane of the membrane,^37,38^*E*_*z*_ = *V*_mp_/*L*_*z*_, where *V*_mp_ is the desired membrane potential, and *L*_*z*_ is the length of the periodic simulation box along the *z*-axis (which is assumed to be perpendicular to the membrane extending in the *xy*-plane). The PMEPot VMD plugin was used to calculate the average electrostatic potential map from simulation.^39,40^ The transmembrane field felt at position **r** is calculated by subtracting the average map at 0 mV from the map calculated with the applied voltage, [ϕ(**r**; *V*_mp_) – ϕ(**r**; 0)].^37^ This quantity can be averaged over the entire plane of the membrane and then divided by *V*_mp_ to yield the one-dimensional dimensionless potential fraction profile, *f*(*z*), representing the coupling of charges to the transmembrane potential along the *z*-axis perpendicular to the membrane.^37^ Results are shown in Section S3

All the MD simulations, with and without an applied electric field, were 1 μs long. Statistical uncertainties on all quantities extracted from the MD simulations were determined using block averages.

**Figure 1:**
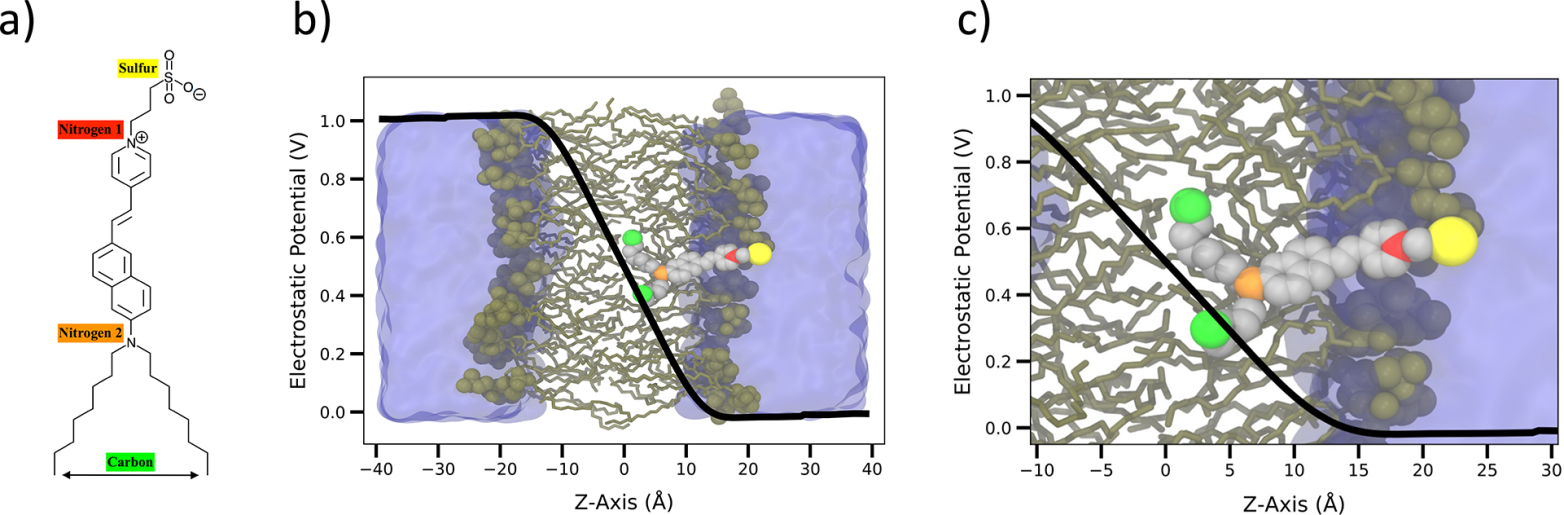
Simulation of di-8-ANEPPS in a DPPC membrane. (a) Schematic depiction of the di-8-ANEPPS molecule with atomic color labeling used hereafter. (b, c) Representative snapshot of di-8-ANEPPS in a solvated DPPC membrane with 1D overlay of a dimensionless fraction of the transmembrane potential (*f*(*z*)).^37^ The dimensionless fraction of the transmembrane potential (*f*(*z*)) was extracted from simulations with the pure DPPC membrane with a series of applied voltages (Figure S2). The oxygens of the sulfate group are not shown in parts b and c for the sake of clarity.

**Figure 2:**
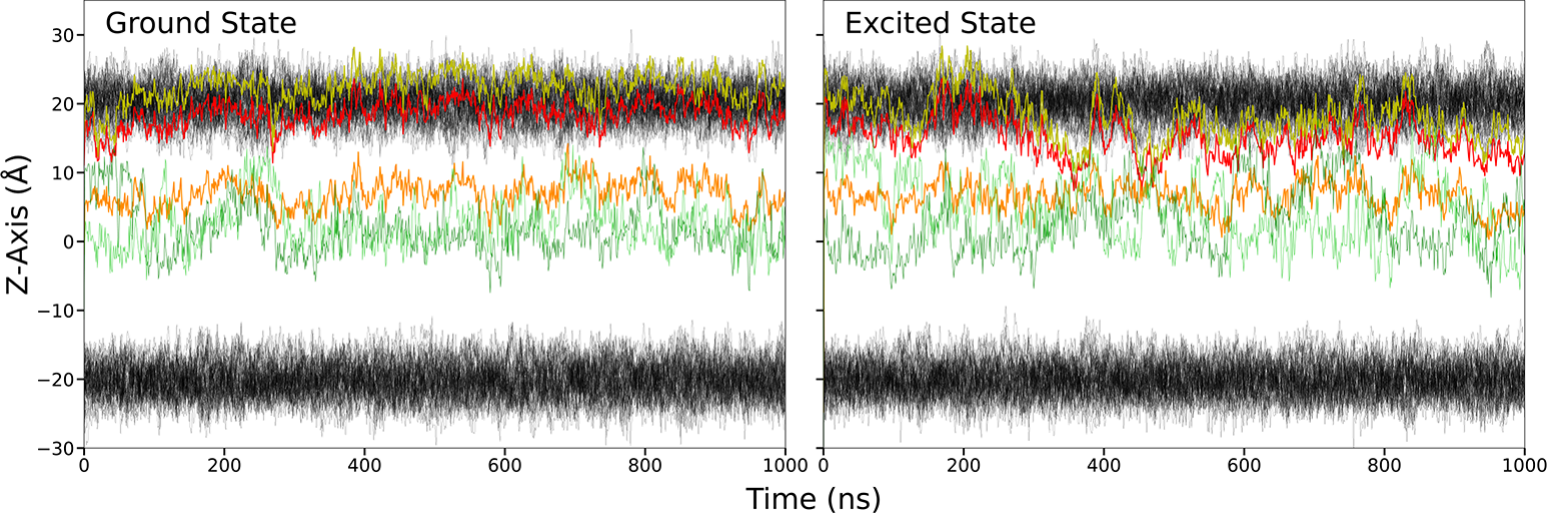
Tracking *z*-coordinates of selected atoms in the ground and excited states of di-8-ANEPPS embedded in a DPPC membrane with no applied voltage. Shown are the sulfur (yellow), the pyridinium nitrogen (red), the amino nitrogen (orange), the last carbon atoms in the hydrocarbon tails (green), and the DPPC lipid head groups (black). The color coding is the same as that in Figure 1.

**Figure 3:**
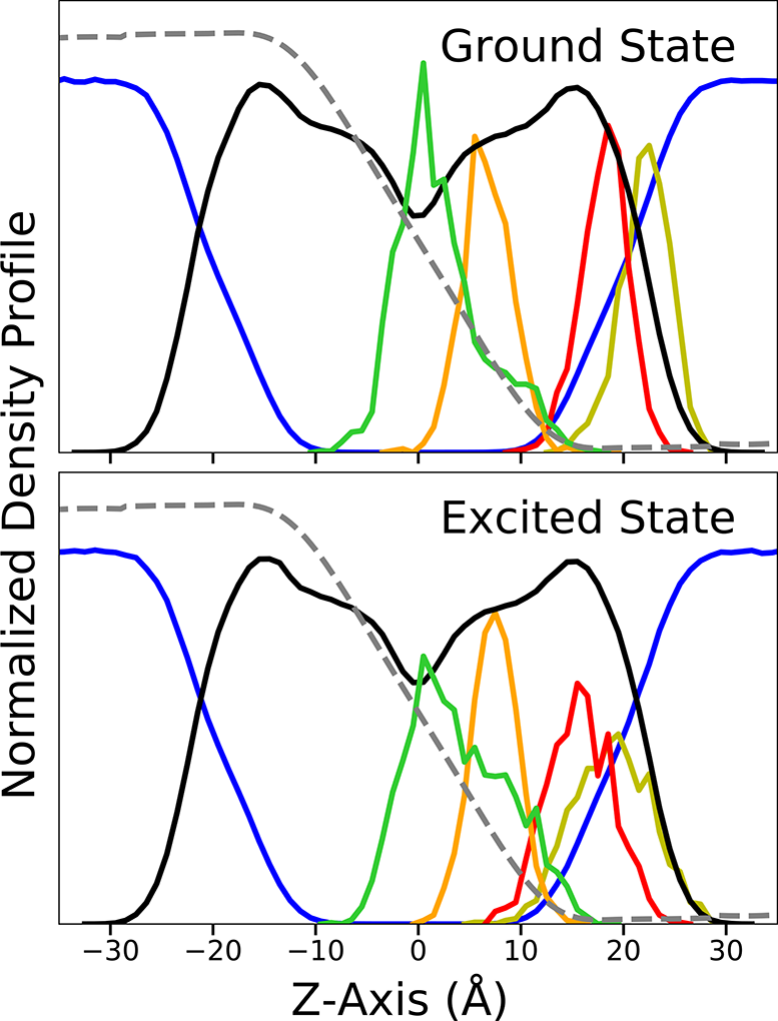
Density profiles of selected atoms of membrane-bound di-8-ANEPPS in the ground and excited states. The membrane potential is 0 mV. The different peaks were scaled relative to one another for clarity. Shown are the sulfur (yellow), the pyridinium nitrogen (red), the amino nitrogen (orange), the last carbon atoms in the hydrocarbon tails (green), the DPPC lipid head groups (black), and the water (blue). The profile of the fraction of the transmembrane potential, *f*(*z*),^37^ is shown in gray.

A typical configuration of di-8-ANEPPS bound to a solvated DPPC membrane is shown in Figure 1. During the simulations, the propyl sulfonate of di-8-ANEPPS remains at the membrane–water interface near the lipid head groups, around +20 Å along the *z*-axis. The two hydrocarbon tails of di-8-ANEPPS reach the midpoint of the bilayer, anchoring the molecule into the membrane. The rigid, conjugated structure composed of the pyridinium, ethylene, and naphthyl ring in the middle portion of the molecule remains within the hydrophobic core of the membrane and interacts infrequently with the lipid head groups when the orientation of di-8-ANEPPS is not parallel with the membrane normal. The positions of a few selected atoms help better characterize the overall position and orientation of membrane-bound di-8-ANEPPS: the sulfur atom of the negatively charged hydrophilic sulfonate group, the pyridinium nitrogen between the conjugated section and the propyl sulfonate, the amino group nitrogen at the opposite end of the conjugated section, and the last carbon atoms in each of the two hydrophobic tails (highlighted on the left of Figure 1). The dynamics of di-8-ANEPPS in the ground and excited states over the course of a 1 μs trajectory in the absence of a membrane potential is shown in Figure 2. While the molecule appears fairly stable when it is in the ground state, it clearly undergoes larger fluctuations when it is in the excited state. Density profiles of the ground and excited states of di-8-ANEPPS in DPPC are shown in Figure 3. Consistent with the time-series shown in Figure 2, the negatively charged sulfonate headgroup remains at the membrane-water interface for both the ground and excited states. The hydrocarbon tails anchor the molecule in the hydrophobic region for both the ground and the excited states. However, while the two terminal carbons of the hydrocarbon tails are typically at a greater depth in the membrane than any other part of the molecule, they undergo transient fluctuations to positions as high as the amino nitrogen at the end of the conjugated portion of the molecule. Interestingly, di-8-ANEPPS tends to lie deeper in the membrane in the excited state than in the ground state on average and undergo larger fluctuations. There are brief periods during which the conjugated ring structure is almost perpendicular to the membrane normal. The molecule can apparently swing up so that it comes closer to the head groups of the DPPC. The two nitrogen on either end of the fused pyridinium and naphthyl rings have a smaller distance between their peaks for the excited state of di-8-ANEPPS than for the ground state, indicating that the conjugated portion of the molecule, which essentially behaves as a single rigid body unit, is tilted relative to the membrane normal. The average tilt of di-8-ANEPPS, defined here as the angle between the N–N vector linking the two nitrogen atoms and the membrane normal, is 25.9° for the ground state and 48.3° for the excited state. When the molecule is more tilted, the positions of the final carbon are correspondingly pulled a bit higher in the membrane. While some membrane-bound chromophores adopt an orientation that is more parallel to the membrane normal,^41,42^ this value is broadly consistent with fluorescence interferometry measurements reporting the angle of the transition dipole moment with respect to the membrane normal as large as 37.8°.^43^ Structurally, the N–N vector provides a reasonable approximation of the direction of the dipole of the di-8-ANEPPS molecule. For example, on average, the dipole of the excited state dipole makes an angle of about 12° with the N–N vector in the excited state simulations, and the dipole vector to the membrane normal increases from about 27–29° for the ground state simulations to about 45–47° for the excited state simulations.

Predicting quantitatively how a membrane potential affects the fluorescence of di-8-ANEPPS is key to better interpreting a wide range of observations about biological membranes. Because the molecule is relatively small and does not significantly disrupt the overall structure of the bilayer, the potential fraction *f*(*z*) derived from the pure DPPC simulations provides a useful starting point to understand the effect of the membrane potential on the fluorescence.^37^ The sulfur is expected to always have the most positive *z*-coordinate, since it typically seeks to interact with bulk water, whereas the two hydrophobic tails reach the greatest depth of the molecule in the membrane. The pyridinium nitrogen (N1) near the propyl sulfonate carries a partial positive charge in the ground state, while the amino group nitrogen (N2) deeper in the membrane core carries a positive charge in the excited state. In Figure 1, *f*(*z*) is overlaid with a picture of di-8-ANEPPS in the membrane, highlighting that the conjugated rings and the amino group nitrogen are the part of the molecule that is the most directly affected by the presence of the membrane potential. The pyridinium nitrogen sits at the edge of the interfacial bulk region, where the potential fraction *f*(*z*) goes to zero (Figure 1c). Nonetheless, the complete coupling of di-8-ANEPPS with the transmembrane potential is complex because the distributions along the *z* axis for the ground and excited states are very broad (Figure 3). To further characterize the system, simulations of membrane-bound di-8-ANEPPS were generated in the presence of a transmembrane potential, with values of −500 mV, −100 mV, 100 mV, and 500 mV.

**Figure 4:**
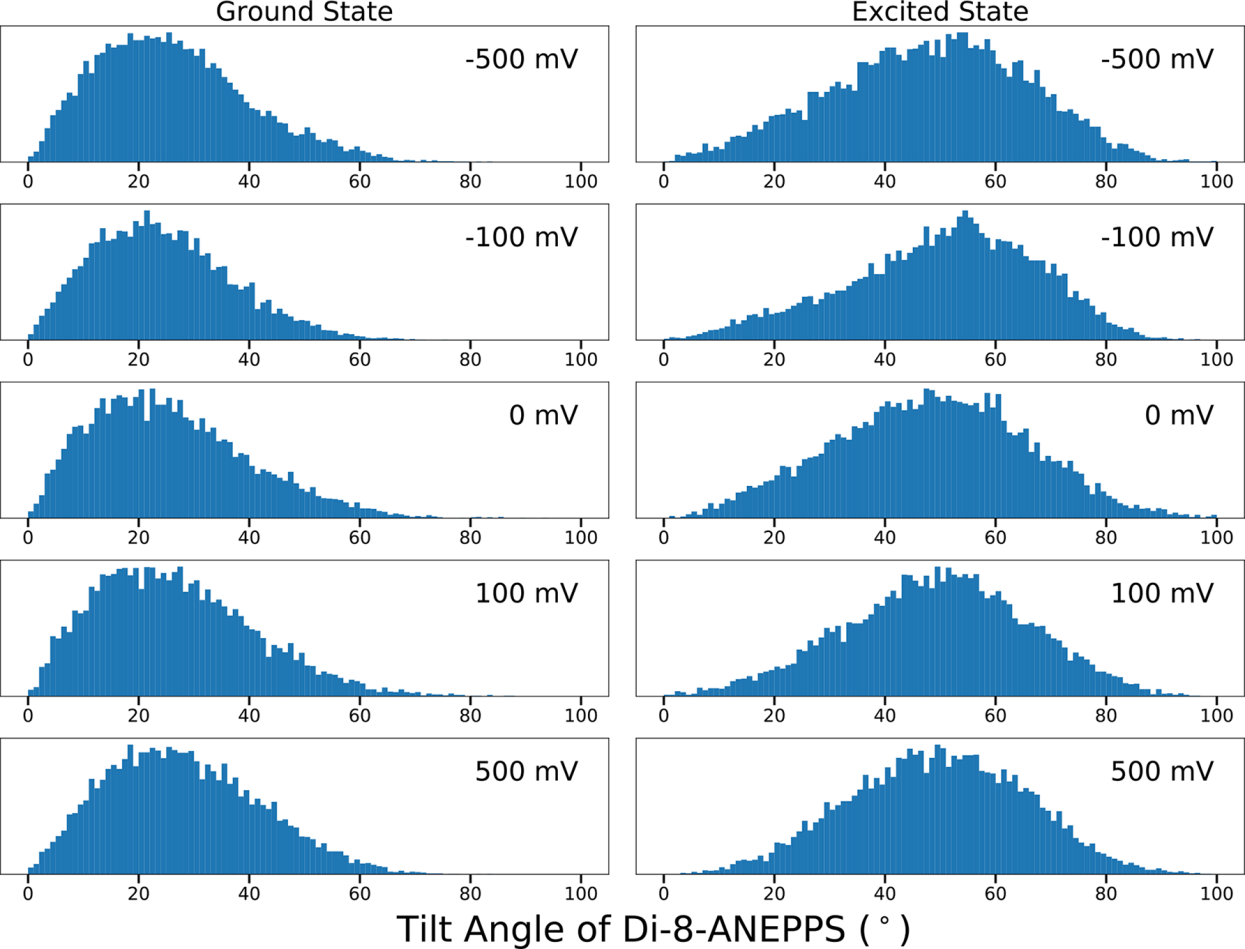
Histograms of the tilt angle between di-8-ANEPPS and the membrane normal over the course of simulations of the ground (left) or excited (right) states. A straight line drawn between the two nitrogen atoms is used to represent the axis of the molecule.

The average position of specific atoms (the sulfur, both nitrogens, and the average of the two terminal carbons) as a function of the membrane potential for the ground and excited states is summarized in Table 1 (Figures S4 and S5 show the time-series tracking the *z*-positions and the corresponding density profiles over the trajectories). Graphs of the average z-positions of each tracked atom for each system with a different applied voltage are included in Figures S6 and S7. The average tilt angles of di-8-ANEPPS in the ground and excited states as a function of voltage are given in the first section of Table 1. Histograms of the tilt angle between di-8-ANEPPS and the membrane normal are shown in Figure 4 (the corresponding time series is shown in Figure S8). For the sake of simplicity, the average tilt of di-8-ANEPPS was defined as the angle between the vector linking the two nitrogen atoms and the membrane normal.

**Table 1:** Average Configuration of Di-8-ANEPPS in DPPC in the Ground (gs) and Excited States (es) as a Function of Membrane Potential Calculated from 10,000 Snapshots Taken over the Course of Each Simulation

	Tilt (deg)		S (Å)		N1 (Å)		N2 (Å)		Carbon (Å)	
*V*_mp_ (mV)	gs	es	gs	es	gs	es	gs	es	gs	es
–500	26.2 ± 13.5	47.9 ± 17.6	21.8 ± 2.5	17.8 ± 3.3	17.9 ± 2.4	14.6 ± 2.9	6.5 ± 2.4	6.3 ± 2.9	2.1 ± 4.4	3.2 ± 5.4
–100	24.5 ± 12.4	50.3 ± 17.0	22.3 ± 2.3	18.0 ± 3.4	18.4 ± 2.2	15.0 ± 3.0	6.7 ± 2.3	7.1 ± 2.5	2.2 ± 4.1	3.8 ± 5.0
0	25.9 ± 14.1	48.3 ± 17.6	22.1 ± 2.5	18.8 ± 3.8	18.1 ± 2.3	15.7 ± 3.2	6.7 ± 2.4	7.5 ± 2.4	2.2 ± 4.3	4.0 ± 4.9
100	26.7 ± 14.1	49.2 ± 16.5	22.2 ± 2.5	18.6 ± 3.5	18.3 ± 2.3	15.6 ± 3.1	7.0 ± 2.3	7.4 ± 2.5	2.4 ± 4.1	3.8 ± 4.7
500	28.2 ± 13.7	49.5 ± 15.9	22.2 ± 2.6	18.7 ± 3.5	18.4 ± 2.4	15.7 ± 3.0	7.2 ± 2.5	7.6 ± 2.3	2.8 ± 4.3	4.3 ± 4.7

For the ground state, the average positions of the tracked atoms appear to be largely insensitive to the membrane potential. This can be explained by the fact that the greatest charge separation is between the locally negative sulfonate and the locally positive pyridinium nitrogen, which are both near the membrane–water interface where the potential fraction *f*(*z*) is close to zero (Figure 1). In contrast, the charge distribution of the excited state spreads over the fused ring portion of di-8-ANEPPS, penetrating the membrane much deeper into the membrane. For this reason, its average position is more sensitive to the transmembrane potential than that of the ground state. With increasing membrane voltage, the di-8-ANEPPS molecule shifts in the positive *z* direction for both the ground and excited states (Table 1 and Figure S6). This is qualitatively consistent with the potential fraction profile, *f*(*z*), shown in Figure 1. Comparing the two extreme voltages applied to the ground state, the range of motion for the sulfur is 0.4 Å for the sulfur, 0.5 Å for the pyridinium nitrogen, 0.7 Å for the amino nitrogen, and 0.7 Å for the final carbons of the tails. The full range of motion is greater for the excited state: 0.9 Å for the sulfur, 0.9 Å for the pyridinium nitrogen, 1.3 Å for the amino nitrogen, and 1.1 Å for the final carbons of the tails. The larger shift shows that the excited state is more sensitive to the membrane potential than the ground state. Though for both the ground and excited states, the molecule straightens out and adopts a deeper position in the membrane at −500 mV. The average tilt angle varies from 26.2° to 28.2° for the ground state and 47.9° to 49.5° for the excited state. The distributions of the tilt angle are very broad, with a root-mean-square deviation on the order of 13–14° for the ground state and 16–18° for the excited state. (Figure 4). Consistent with the present results, a previous MD study reported an angle of 30.7 ± 15.3 for the ground state di-8-ANEPPS in a DPPC membrane (measured between the vector of the two nitrogen and the membrane normal).^22^

In accord with the Franck–Condon principle, the movement of electrons occurs on a much shorter time scale than any nuclear motion and thus the electronic transition can be considered without any change in nuclei position.^44−46^ In the present classical trajectories, the electronic transitions between the ground and excited states are simulated by switching between the force fields representing the ground and excited states. To determine the absorption energy, the total energy difference of the system, *ΔE*_ab_ = *E*_es_ – *E*_gs_, is calculated for each configuration taken from a trajectory of di-8-ANEPPS in its ground state. Similarly, to determine the emission energy, the total energy difference of the system, *ΔE*_em_ = *E*_es_ – *E*_gs_, is calculated for each configuration taken from the trajectory of di-8-ANEPPS in its excited state. The peak absorption wavelength is given by λ_ab_ = *hc*/⟨*ΔE*_ab_⟩_(gs)_, where the average is taken over a trajectory of di-8-ANEPPS in its ground state, and the peak emission wavelength is given by λ_em_ = *hc*/⟨*ΔE*_em_⟩_(es)_, where the average is taken over a trajectory of di-8-ANEPPS in its excited state.

Dynamical relaxation effects in the fluorescence could be examined by monitoring the change in the energy difference as a function of time after switching the force fields, modeling the ground and excited states. To account for the energy contribution due to nonradiative vibrational transitions, two voltage-independent empirical offset constants were incorporated into the calculation of the absorption and emission transitions energies, optimized to best match the experimental data following a procedure similar to what was previously done for di-8-ANEPPS in solvents of various polarity.^23^ Such a treatment, entirely based on classical trajectories and molecular mechanical force fields, does not account for nonradiative intramolecular transitions associated with the reorganization of the nuclear configuration of the molecule caused by differences in the ground state and excited state potential energy surfaces.^47−49^ Advanced QM treatment generally considers the curvature of the initial and final state potential energy surface and the corresponding intramolecular vibrational states to account for the state-dependence of the energy minima and its coupling to the electronic transition.^49−52^ For the sake of simplicity, these effects in the present work are entirely subsumed into environment-independent empirical offset constants for the excitation and emission.^53,54^ This approximation is justified, as our goal is to investigate the effect of the environment on the absorption and emission energy of membrane-bound di-8-ANEPPS, specifically the effect of the membrane potential.

The offset for emission energy difference was optimized to be 12.6 kcal/mol using the data from Kao et al. for di-8-ANEPPS in membrane.^55^ While this value is slightly larger than the emission offset previously determined for di-8-ANEPPS in solvents (10.0 kcal/mol),^23^ the change has no impact on the following analysis. The emission wavelengths reported in this work use an offset based on the value extracted from the emission wavelength with maximum intensity from Kao et al.^55^ Because no data is available for the voltage-dependent absorption wavelength of di-8-ANEPPS in membrane, the energy offset of 4.99 kcal/mol (previously determined for absorption of di-8-ANEPPS in solvents of different polarity)^23^ was used here as the absorption offset constant. Accounting for internal relaxation, the QM reorganization energies are expected to be on the order of about 1,000 cm^–1^ (2.9 kcal/mol) for one state. Assuming simple shifted harmonic potential energy surfaces, the difference between the absorption and emission frequency is expected to be twice the reorganization energy, thus, the two offset constants of a fluorescent molecule are expected to differ by about 6 kcal/mol. The difference between the empirical offset constants is reasonably close to this estimate.

**Figure 5:**
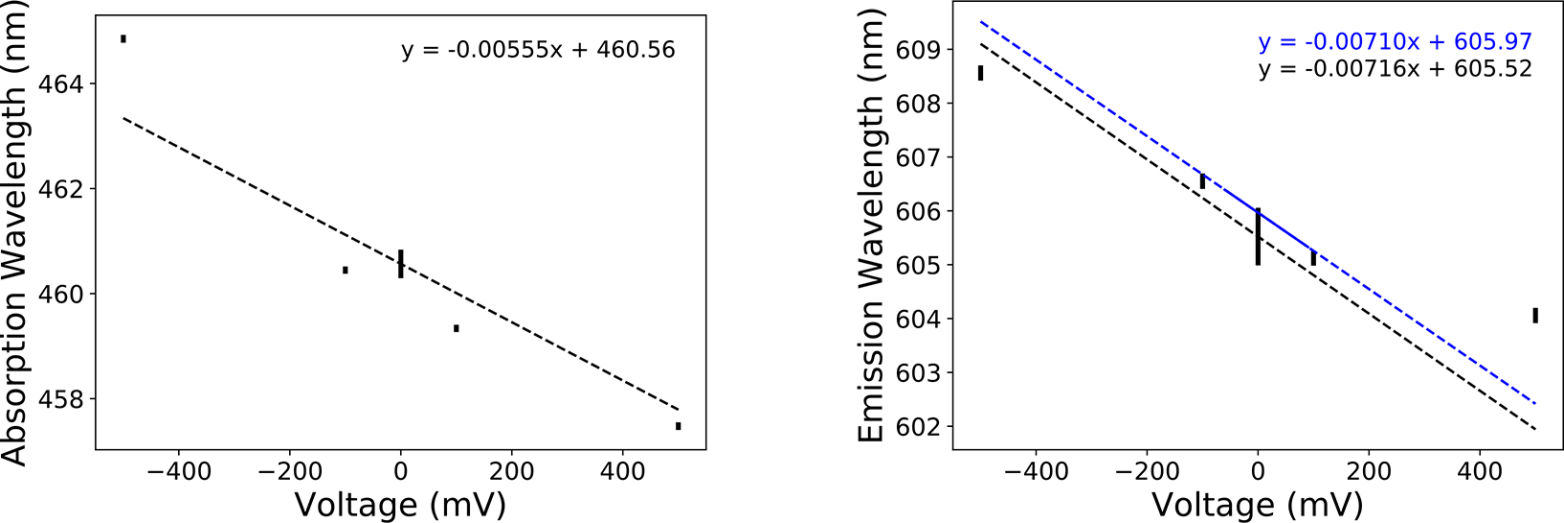
Absorption (left) and emission (right) wavelengths for di-8-ANEPPS in DPPC with a range of applied voltages. The trendline of the di-8-ANEPPS absorption and emission data is fitted on the basis of the −100 to 100 mV range and is plotted in black. The emission wavelengths trendline deduced from fitting a Lorentzian to the data of Kao et al. of range −60 to +90 mV^55^ (λ_max_ = 605.97–0.00710 *V*_mp_) is in blue. The statistical uncertainties on the values extracted from MD simulations were estimated by using block averages. For the absorption, the uncertainties are on the order of ±0.07 nm for all values and ±0.3 nm for the simulation at 0 mV. For the emission, the uncertainties are on the order of ±0.14 nm for all values and ±0.5 nm for the simulation at 0 mV.

Direct comparison of the calculated absorption and emission wavelengths with the experimental data requires some additional analysis. Experimental studies reporting changes in fluorescence of such membrane-bound probe molecules do not typically report the shift in the peak of the absorption or emission wavelengths as a function of membrane potential, but commonly rely on dual-wavelength fluorescence ratiometry.^55,56^ Dual-wavelength ratiometry is a procedure by which the fluorescence intensity is measured using two wavelengths and reported as the percent change in the ratio of those two values with respect to voltage. These two wavelengths could refer to either excitation or emission wavelengths. In the case of excitation ratiometry, excitation is performed at two different wavelengths, and the fluorescence intensity at a single emission wavelength is recorded for each. For emission ratiometry, a single excitation wavelength is used and measurements are made of the fluorescence intensity at two different emission wavelengths.^3,7,57^ One advantage of reporting results in this way is that this term is insensitive to the dye binding at different locations and insensitive to the specific concentration of the dye molecule in a given preparation.^58^ The concept of performing dual wavelength ratiometric measurements was utilized with fluorescent cation detectors^59^ and potentiometric indicators,^60^ before being applied to a probe whose spectra shifts in response to membrane potential.^56^ Emission ratiometry was shown to be a viable means of detecting transmembrane potential caused by externally applied electric field with di-8-ANEPPS,^61^ but has been shown to be less effective when investigating intramembrane electric field strength due to membrane dipole potential and fluidity.^7^ Using the data of Kao et al.,^55^ who measured the ratio of emission intensity of di-8-ANEPPS in Human Embryonic Kidney (HEK) cells at wavelengths of 620 and 560 nm for membrane potentials ranging from −60 to +90 mV, we sought to determine the actual shift in the emission wavelength as a function of membrane voltage. A standard Lorentzian line shape function was fitted to the experimental emission spectra (Figure 5 in Kao et al.).
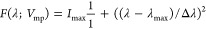
1Here *I*_max_ = 153.4 is the height of the peak, *Δλ* = 51.1 nm is the width of the peak, and λ_max_ = 605.97–0.0071 *V*_mp_ nm is the voltage-dependent position of the peak. While other functional forms could be used, this line shape matches the experimental data and is adequate for the purpose of converting the reported ratio emission at two wavelengths into a shift of the maximum wavelength. The functional form reproduces the voltage-dependent shift from Kao et al. (bottom of Figure 3 in Kao et al.), such that the relative change *F*(620; *V*_mp_)/*F*(560; *V*_mp_) – *F*(620; −100 mV)/*F*(560; −100 mV) matches the linear relationship of −0.0352 to 0.000352 *V*_mp_ measured experimentally. The value of *I*_max_ does not affect the voltage dependence. The optimized Lorentzian line-shape and the reproduction of the data from Kao et al. for the change in fluorescence ratio as a function of voltage are shown in Figure S9 along with a more in-depth description of the procedure used to create them. On the basis of this analysis, the calculated emission shifts produced from the simulations of this work can be directly compared with the data from Kao et al.^55^ The results for absorption and emission are given in Table 2, together with the estimates extracted from the experimental data. Without any applied voltage, the absorbance wavelength is 460.6 nm which is quite close to the values reported in other membrane systems. For example, in muscle fibers, it was recorded to have an absorption peak at 470 nm.^6^ The calculated absorption and emission wavelengths as a function of membrane potential are plotted in Figure 5.

**Table 2:** Absorption, Emission and Stokes Shift of Di-8-ANEPPS in a DPPC Membrane (nm)^a^

Voltage (mV)	Absorption	Emission	Stokes Shift
–500	464.8507316	608.5598257 (609.5)	143.709094
–100	460.4470742	606.5502935 (606.7)	146.1032194
0	460.5651479	605.5221392 (606.0)	144.9569914
100	459.3371888	605.119289 (605.3)	145.7821002
500	457.4763198	604.0569262 (602.4)	146.5806064

a Number in parentheses were deduced from fitting a Lorentzian to the data of Kao et al.^55^

As observed in Figure 5, a linear relationship can be drawn for the emission as a function of the externally applied membrane potential (n.b., the statistical uncertainties for the average values extracted from the MD trajectories inscribed in the figure are essentially negligible). With increasing voltage potential across the membrane, there is a slight but noticeable decrease in the peak fluorescence predicted by this model. In the full range of data produced by this study, from −500 to 500 mV: for every 100 mV, the wavelength of the fluorescence is shown to change by an average of 0.45 nm. However, if the range is narrowed to the interval of −100 to 100 mV, the wavelength of the fluorescence is shown to change by an average of 0.716 nm for every 100 mV (shown in the right side of Figure 5 as the black trendline). This slight discrepancy may be due to the larger effect of larger membrane potential, resulting in the change no longer being linear at those values of applied voltages. The difference may seem minor, but the linear relationship derived from the Lorentzian fit of the spectra of Kao et al. predicts a change of 0.710 nm per 100 mV^55^ (shown on the right side of Figure 5 as the blue trendline). Kao et al. tested a range of only −60 mV to +90 mV, which is somewhat smaller than the ranges others typically test, for example −280 mV to 140 mV in cardiac cells^62^ and 0 mV to 250 mV in spherical lipid bilayers.^63^ Both of these studies showed linearity of the fluorescence ratio over the tested range of voltages for their probe molecules, which were di-8-ANEPPS and di-4-ANEPPS respectively. It can be seen in this comparison that the fluorescence data produced by this work are consistent with at least the experimental results of Kao et al. in the reduced range of voltages. Other studies concluded that di-8-ANEPPS has a very linear response to transmembrane potential in a physiological range of −280 to +140 mV, as was shown in a calibration of di-8-ANEPPS when simultaneously comparing the optical and electric measurements during voltage clamp. And notably it was also found in that work that this linearity was not maintained above or below that range.^62^ As shown by these experimental studies,^55,62,63^ the ±500 mV simulations performed in the present work are well outside of the linear response range. Hence only the data produced by the simulations in the ±100 mV range were used for the linear fit shown in Figure 5.

The present study demonstrates the feasibility of quantitatively modeling the fluorescence of a probe like di-8-ANEPPS in a membrane system and opens up the possibility of predicting the changes in its reporting in more complex environments simulated with MD simulations. An important measure of the success of the present simulations is provided by Figure 5, showing the linear relationship between the peak emission wavelength and the membrane voltage. The agreement between the calculation and the voltage-dependent emission data from Kao et al.^55^ is excellent, as long as the magnitude of the applied voltage does not exceed 100 mV. Considering that the calculated observable depends on the force fields for both the ground and excited states, as well as the remainder of the system, the good agreement with experiment is indicative that the atomic models and simulation methodologies are sound. Unfortunately, there are no experimental data allowing a similar comparison for the absorption data as a function of membrane potential at this point. Future work based on this simulation approach shall explore the effects of diverse factors such as membrane composition,^9,13^ ion concentration,^14,15^ osmotic pressure,^16^ and cholesterol.^17^

## Supporting Information Available

The force field parametrization of di-8-ANEPPS, membrane potential profiles, tracked positioning of key atoms of di-8-ANEPPS over the course of simulation, and details on how experimental data was handled for direct comparison (PDF)
Transparent Peer Review report available (PDF)
