# Supplementary material for: Simulating the Voltage-Dependent Fluorescence of Di-8-ANEPPS in a Lipid Membrane
Source: J Phys Chem Lett. 2023 Sep 7;14(36):8268–76. doi: 10.1021/acs.jpclett.3c01257 (PMC10510438; doi:10.1021/acs.jpclett.3c01257)
Supplement: Supplementary file 1 — jz3c01257_si_001.pdf [file jz3c01257_si_001.pdf]

Supporting information for: Simulating the Voltage-Dependent Fluorescence of Di-8-ANEPPS in a Lipid Membrane

Rachael Youngworth† and Benoît Roux*,‡

†*Department of Chemistry, The University of Chicago, 5735 S Ellis Ave, Chicago, IL 60637, Chicago, IL 60637, USA*

‡*Department of Biochemistry and Molecular Biology, The University of Chicago, 929 E. 57th Street W225, Chicago, IL 60637, U.S.A.*

E-mail: roux@uchicago.edu

1) Force field parameterization of di-8-ANEPPS

The GAAMP (General Automated Atomic Model Parameterization) web server[S1] was used to generate nonpolarizable and polarizable force fields for the ground state of di-8-ANEPPS. Geometry optimizations were performed at the HF/6-31G* level and the electrostatic potential calculations for the polarizable model used B3LYP/aug-cc-pVDZ. This program is based on improving the partial charge assignments from either CGenFF (CHARMM General Force Field)[S2] or GAFF (general Amber force field) by fitting to electrostatic potential data from QM calculations and by fitting to the results of compound-water interaction tests. The torsion potentials were also optimized by fitting to the conformer energies resulting from a one-dimensional dihedral scan. Because the important and more unique characteristics of the probe are entirely contained within the head group and fused ring structure, a slightly shorter version of di-8-ANEPPS (di-4-ANEPPS) was parameterized directly with GAAMP to reduce the number of soft, flexible dihedrals that would need to be considered. The hydrocarbon chains were restored afterwards with parameters taken from saturated lipid tails (found in the CHARMM toppar files[S2]) that were then connected to the ends of the previously cleaved chains. This same method was used for the excited state extension from di-4-ANEPPS to di-8-ANEPPS as well, since the properties at the ends of the alkane chains should not display any electronic changes during absorption or emission. The excited state requires the generation of QM electrostatic potential data specifically representative of the desired state, and GAAMP's charge fitting procedure was used once the electrostatic potential data was generated.

Di-8-ANEPPS has been studied before with CASSCF methods and simulated with mixed QM-MM methods.[S3,S4] The full pi system consists of 20 electrons and 19 orbitals, but the computational study from Robinson et al. shows that the transition to the first excited singlet state can be fully represented by a CASSCF(6,6) (where 6 electrons and 6 orbitals are considered to be the active space) with a fragment of the full molecule.[S3] Due to the transition from ground to excited states for di-8-ANEPPS involving reorganization of charge over the conjugated ring, its active molecular orbitals largely concentrate their occupancy in pz atomic orbitals on those carbon (assuming the molecule lies in the $x - y$ plane). Because of this, it was easiest to recognize the molecular orbitals

of interest by starting the molecule flat in the $x - y$ plane so that the desired reported occupancies were entirely of pz-character. Then the orbitals that are initially placed in the active space without any z-character can be rotated out in favor of the desired orbitals before continuing to converge the CASSCF calculation. Initially attempts were made to replicate these orbital occupancies for a truncated structure consisting of only the active space of the molecule (the rigid ring structure spanning from nitrogen to nitrogen without the propyl sulfonate or the octane chains) used in a study by Robinson et al.[S3] Ultimately convergence Replicating this CASSCF calculation for the truncated structure was achieved at the def2-SVP def2-SVP/C level with orbstep SuperCi and switchstep DIIS convergence criteria included using ORCA[S5,S6] The results of the CASSCF calculation of the isolated first excited singlet reflect the excited state after an instantaneous electronic transition from the ground state, meaning that the geometry of the molecule has not been updated since the initial ground state geometry optimization. The molecular orbitals of that active space are included as Supplemental Figure S1. Successfully characterizing the truncated molecule did

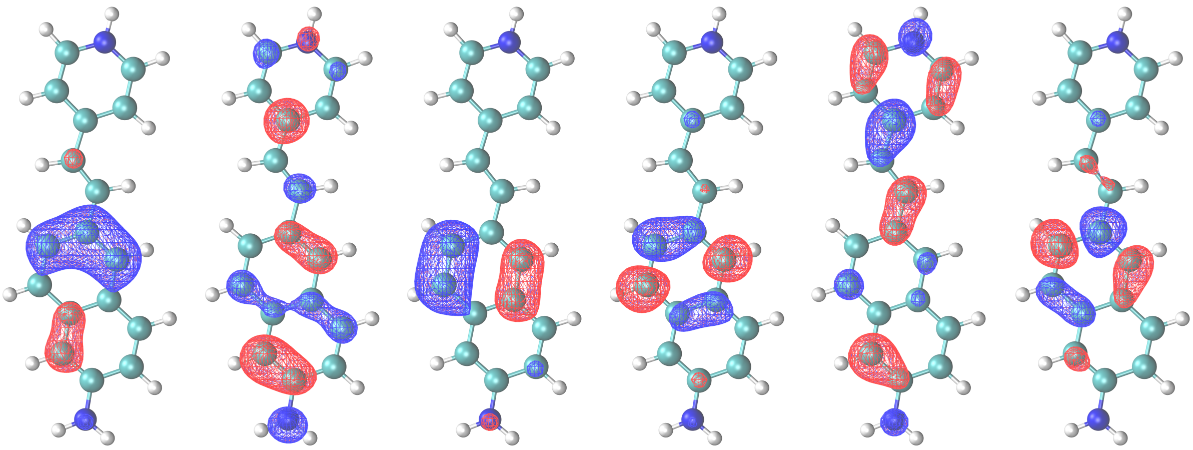

Figure S1: Active orbitals of the truncated di-ANEPPS fragment - converged to through CASSCF calculations. The occupancies from left to right are: 1.94, 1.47, 1.92, 0.09, 0.53 and 0.06 (which are quite similar to those found by Robinson et al. for the first excited state: 1.94, 1.40, 1.90, 0.10, 0.60, 0.06).[S3]

not allow for an easy transition to utilizing the tools of GAAMP to convert the overall electrostatic potential surrounding the molecule to partial charges on each of its atoms, especially since this cationic fragment does not account for contributions from the sulfonate that neutralizes the overall

molecule. Additionally defining the amino as primary rather than tertiary affects the treatment of all dihedrals that include it and also affects the amino nitrogen's accessibility when calculating its affinity as a hydrogen bond acceptor. Repeating the procedure of calculating the QM electrostatic potential for di-4-ANEPPS (which unlike the truncated fragment treats the amino nitrogen as tertiary rather than primary and includes the contributions from the sulfonate that neutralize the molecule) was facilitated by rotating in the orbitals known to be in the active space for the truncated structure into the active space of this molecule. The orbitals of the active space for the excitation of di-4-ANEPPS to its first excited singlet state are shown in Figure S2. Each orbital in the ac-

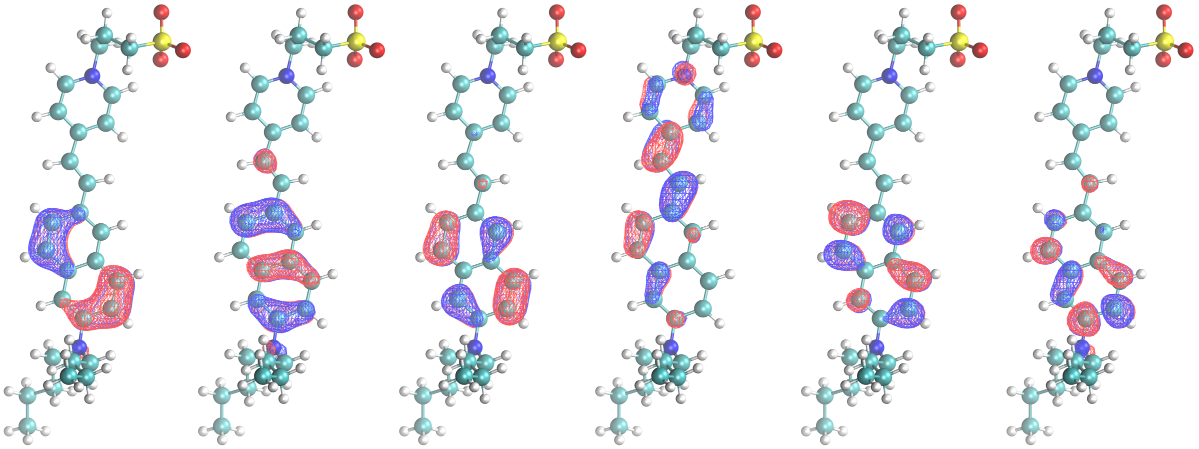

Figure S2: Active orbitals of di-4-ANEPPS - converged to through CASSCF calculations. The occupancies from left to right are: 1.92, 1.82, 1.14, 0.89, 0.17, 0.06. This shows that the excited state is largely characterized by the excitation of an electron from the third orbital (reducing its occupancy to 1.14) to the fourth orbital (increasing its occupancy to 0.89).

tive space is considered to have partial occupancy rather than either being fully occupied with two electrons or empty. In this calculation, six orbitals are considered that share a total of six electrons. The first three orbitals have occupancies approaching two while the rest have occupancies closer to zero. While the ground state would essentially have either full or empty orbital occupancy, the excited state shows decreased occupancies in the previously fully occupied orbitals and increased occupancies for the previously vacant orbitals. This calculation shows that the transition to the first excited state of di-4-ANEPPS largely involves a shift in occupancy from the third orbital to the fourth orbital of the active space shown in Figure S2. GAAMP's electrostatic potential fitting

protocol was applied to the excited state using the electrostatic data generated with CASSCF for the di-4-ANEPPS structure.

The truncated structure of di-8-ANEPPS was found by Robinson et al. to have a ground state dipole of 14.9 Debye and an excited state dipole of 3.4 Debye via a CASSCF(6,6) calculation.[S3] The replication performed with a CASSCF(6,6) calculation in the present work resulted in dipoles of 17.28 Debye and 4.56 Debye for the structure in its ground and excited state respectively. Another QM/MM study based on the full structure reported a ground state dipole of 36.7 ± 2.7 Debye and an excited state dipole of 48.3 ± 2.6 Debye (a change of 11.4 ± 1.7 Debye).[S4] Using CASSCF(6,6) for the di-4-ANEPPS structure, dipoles of 22.34 and 33.14 Debye for the ground and excited state respectively were found, which exhibits a very similar change of 10.80 Debye. After generating parameter files with GAAMP based on this data and converting it to di-8-ANEPPS, the change in dipole is largely maintained. The parameter set produces dipoles of 31.23 Debye and 41.40 Debye for the ground and excited state, which is a change of 10.17 Debye. The force fields for di-8-ANEPPS in its ground and LE excited states parameter files are given in https://github.com/RouxLab/Fluorescence-of-Di-8-ANEPPS.

2) Membrane potential profiles

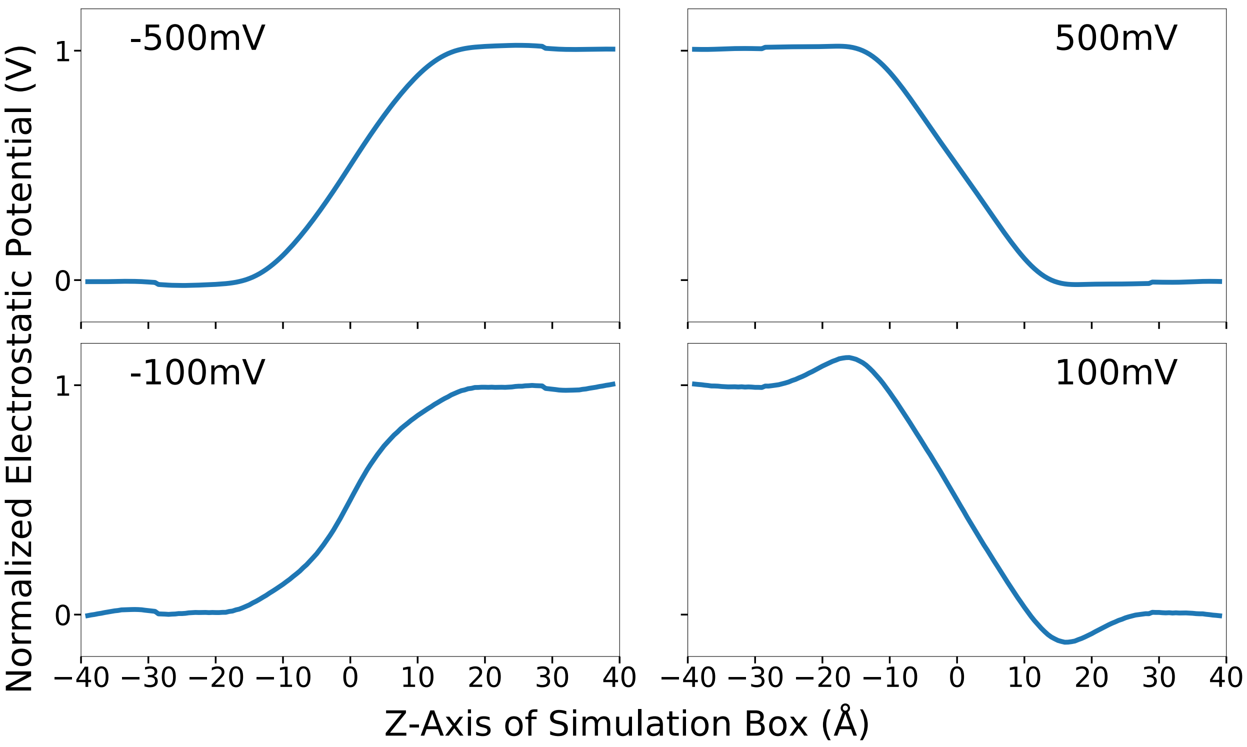

Figure S3: 1D normalized ($\phi(z)/V_{\text{mp}}$) PMEPot graphs along the z-axis of a pure DPPC membrane with applied voltages - after subtracting out the 0 mV PMEPot data

3) Times-series and density profiles for selected atoms

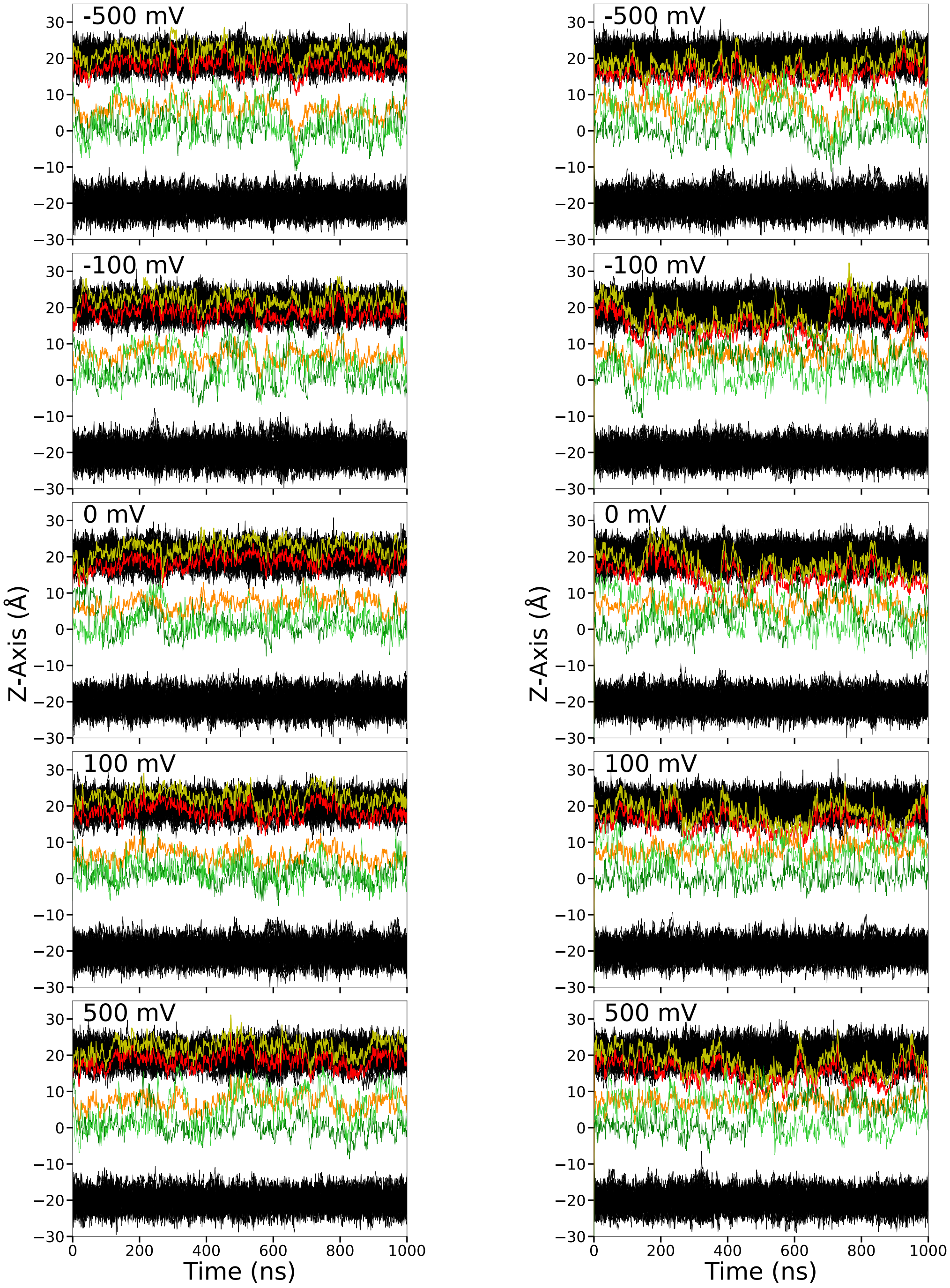

Figure S4: Tracking z-coordinates of the selected atoms in the ground (left) and excited (right) states of di-8-ANEPPS embedded in a DPPC membrane with a range of applied voltages - yellow is the sulfur, red is the pyridinium nitrogen, orange is the amino nitrogen, the two green shades are the final carbons in the octane chains, and black is the DPPC lipid head groups

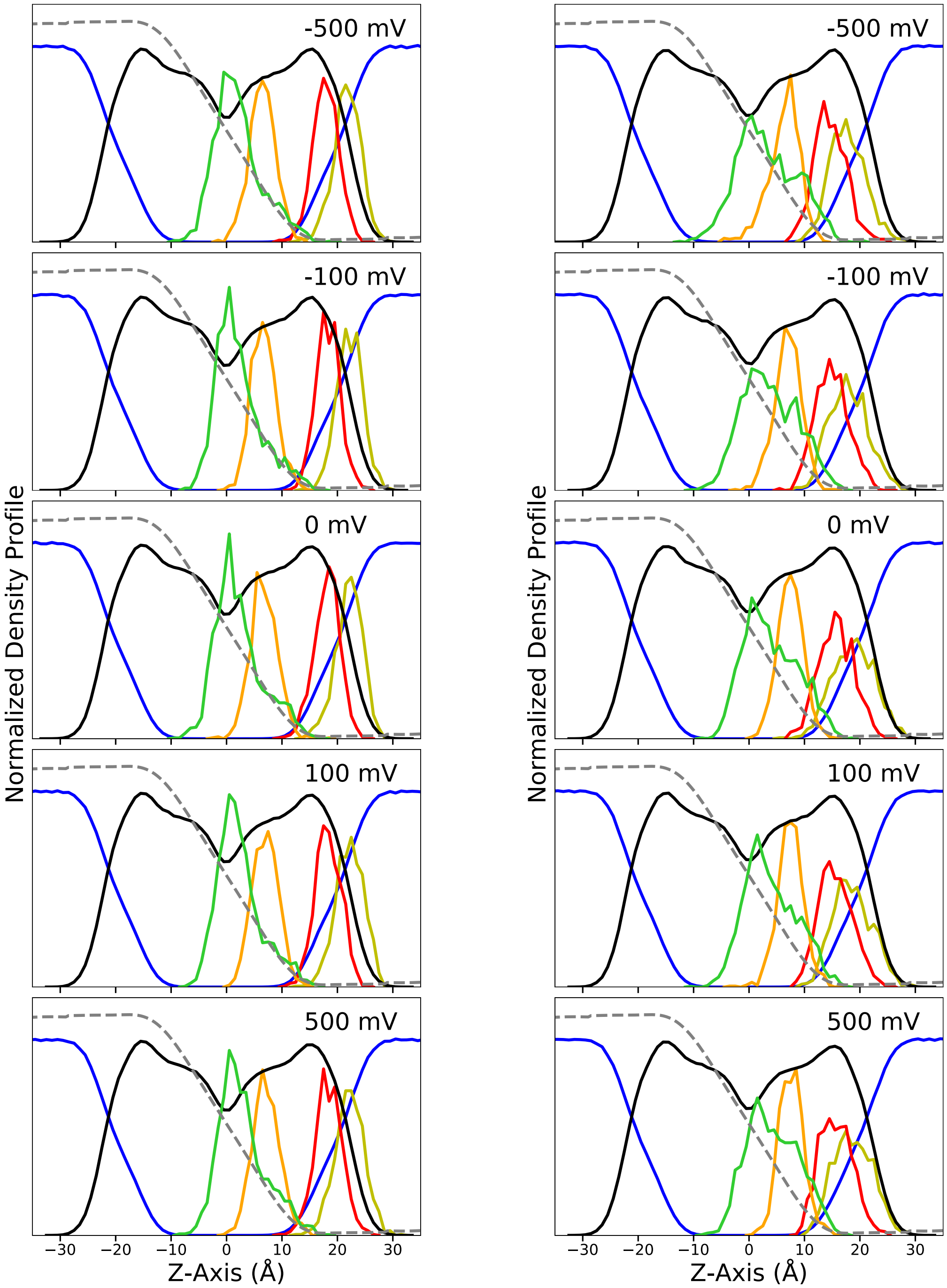

Figure S5: Density profiles of the selected atoms of the ground (left) and excited states (right) of di-8-ANEPPS in DPPC and water with a range of applied voltages, scaled relative to one another for clarity - yellow is the sulfur, red is the pyridinium nitrogen, orange is the amino nitrogen, green is the average of the final carbon in the octane chains, black is the DPPC lipids and blue is water - with 1D overlay of a normalized ($\phi(z)/V_{\mathrm{mp}}$) transmembrane potential in gray

3) Average positions and tilt

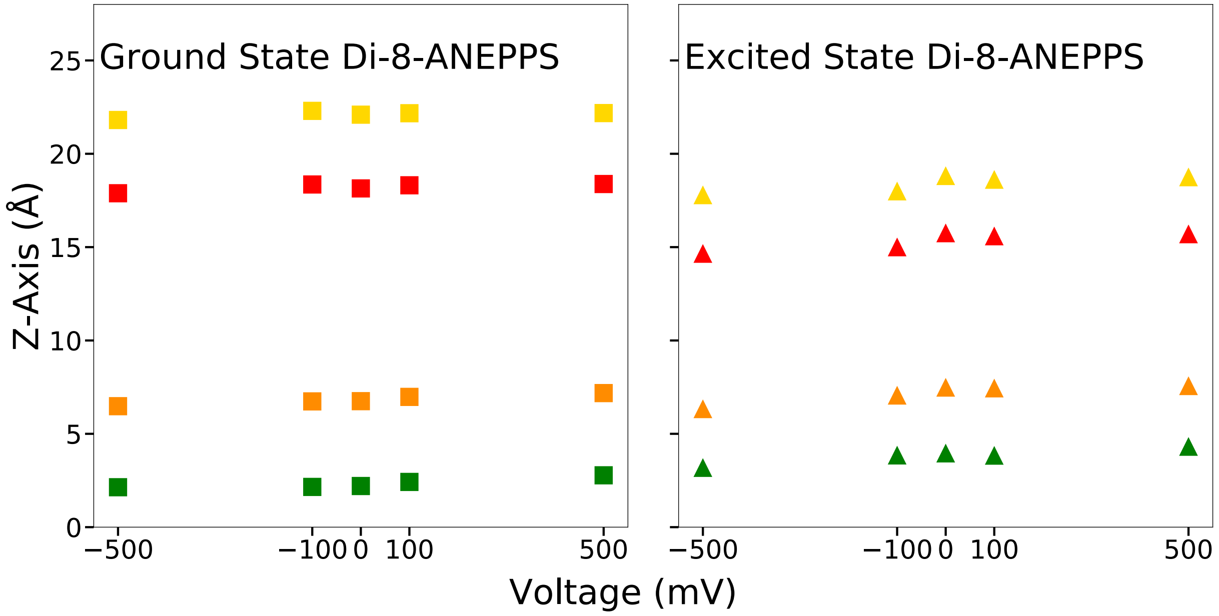

Figure S6: Z-coordinates of the selected atoms in the ground and excited state di-8-ANEPPS in DPPC with a range of applied voltage

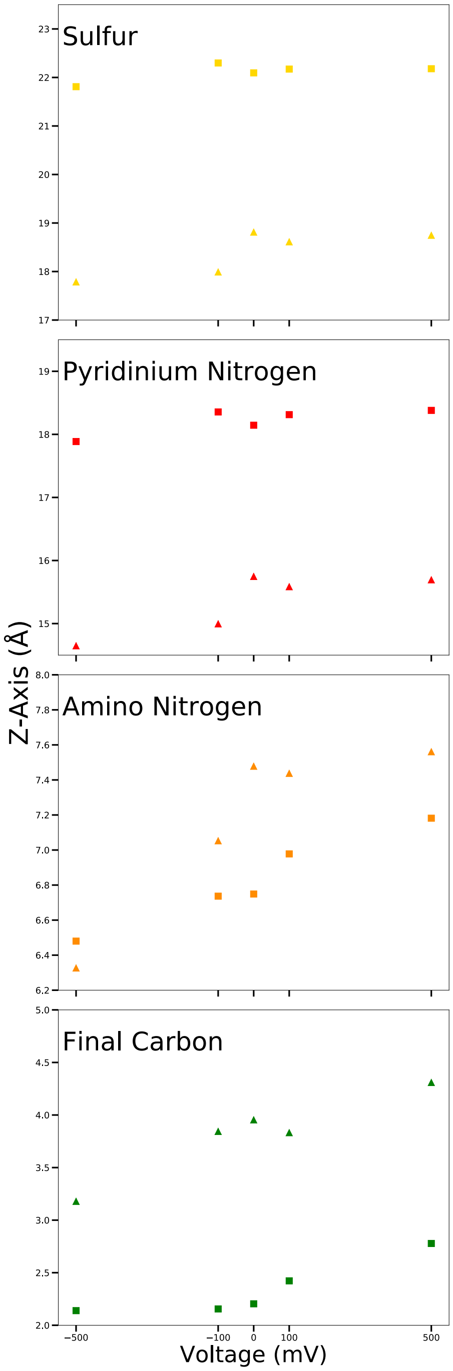

Figure S7: Z-coordinates of the selected atoms in the ground (squares) and excited (triangles) state di-8-ANEPPS in DPPC with a range of applied voltage

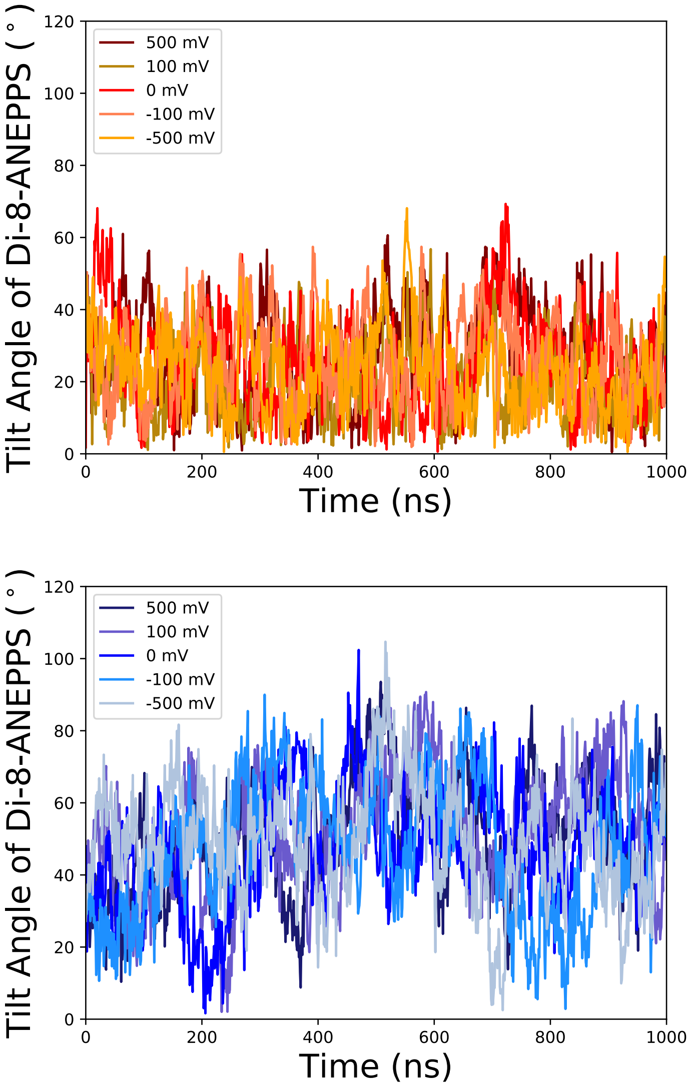

Figure S8: Tilt angle between di-8-ANEPPS and the membrane normal over the course of simulations of the ground (top) or excited (bottom) states. A straight line drawn between the two nitrogen represents the axis of the molecule.

4) Experimental data

A relative fluorescence change per 100 mV for di-4-ANEPPS was found to be $9.52 \pm 0.02\%$ in A-431 cells, $8.6 \pm 1.4\%$ in rye protoplast, $3.7 \pm 2.0\%$ in a fungus spore, and $12.0 \pm 1.5\%$ in RBL cells.[S7] Additional studies reported a change of 9% per 100 mV in a spherical lipid bilayer,[S8] 9.5% per 100mV in HeLa cells,[S9] up to 9% due to action potential amplitude (which is typically about 100 mV) in rat myocardium,[S10] and 1% per 100mV in rat superior cervical ganglion.[S11]

Many studies of di-8-ANEPPS rely on excitation ratiometry, which is a method based on measuring the fluorescence intensities at a single wavelength that result from excitation performed at two distinct wavelengths and reporting that ratio. The data produced by the work presented here is only the wavelength of either absorption or fluorescence at their peak intensity and it keeps the excitation and emission processes independent, meaning that there is no way to link a given excitation wavelength to the expected intensity of the corresponding fluorescence. However emission ratiometry can also be performed for a system containing di-8-ANEPPS where only a single wavelength was used for excitation and the ratio is instead between detected intensities at two emission wavelengths.[S12] In order to compare to the data of Kao et al., the fluorescence wavelength at peak intensity needs to be derived from their reported change in intensity ratios. Taking the emission spectra graphed in that paper, the intensity peak was fitted with a Lorentzian curve. This functional form, which matches well the published data, was mainly used to convert the reported ratio emission at two wavelengths into a shift of the maximum wavelength (which was not reported). Other similar lineshape functions might have been used as long as they reproduce the experimental data.

$$F(\lambda) = I_{\max} \frac{1}{1 + \big((\lambda - \lambda_{\max})/\Delta\lambda\big)^2} = 153.4 \frac{1}{1 + \big((\lambda - \lambda_{\max})/51.1\big)^2} \tag{1}$$

where $\Delta\lambda = 51.1$ nm is half the width at half the maximum of the curve, $I_{\max} = 153.4$ is the the height of the intensity and $\lambda_{\max} = 605.97 - 0.00710\,V_{\mathrm{mp}}$ nm is the wavelength that produces the peak intensity. When different voltages are applied, the spectral curve is shifted only along the $x - axis$ (changing wavelengths that correspond to a given position on the curve). The

shape of the curve does not change, meaning that the specific value of $I_{\max}$ does not affect the voltage dependence. The predicted intensity along the Lorentzian curve is then determined at two wavelengths (620 nm and 560 nm) and the ratio between these two values can then be graphed against the voltage that was applied to the system to produce that emission peak. These ratios are reported by Kao et al. as a change in ratio (relative to the ratio reported at -100 mV) versus the voltage applied. Reproducing that linear relationship with the Lorentzian drawn based on their spectral curve results in:

$$\Delta(F_{620}/F_{560}) = \frac{F(620; V_{\mathrm{mp}})}{F(560; V_{\mathrm{mp}})} - \frac{F(620; -100\mathrm{mV})}{F(560; -100\mathrm{mV})} = -0.000352\, V_{\mathrm{mp}} - 0.0352 \qquad (2)$$

By matching the ratio data from Kao et al. with the knowledge of the shape of the emission curve, the degree to which that curve is shifted as a function of voltage is determined. This means that the wavelength at the max intensity can now be derived for a given applied voltage from their data. A linear relationship between an applied voltage and the emission wavelength at the intensity peak can be assumed, where the y-intercept is the wavelength at the intensity peak when no voltage is applied and the slope is the optimized term for the shift of the curve as a function of voltage.

$$\lambda_{\max} = 605.97 - 0.00710 V_{mp} \qquad (3)$$

The derived emission wavelengths at peak intensity at different applied voltages can then be directly compared to the results produced in this work.

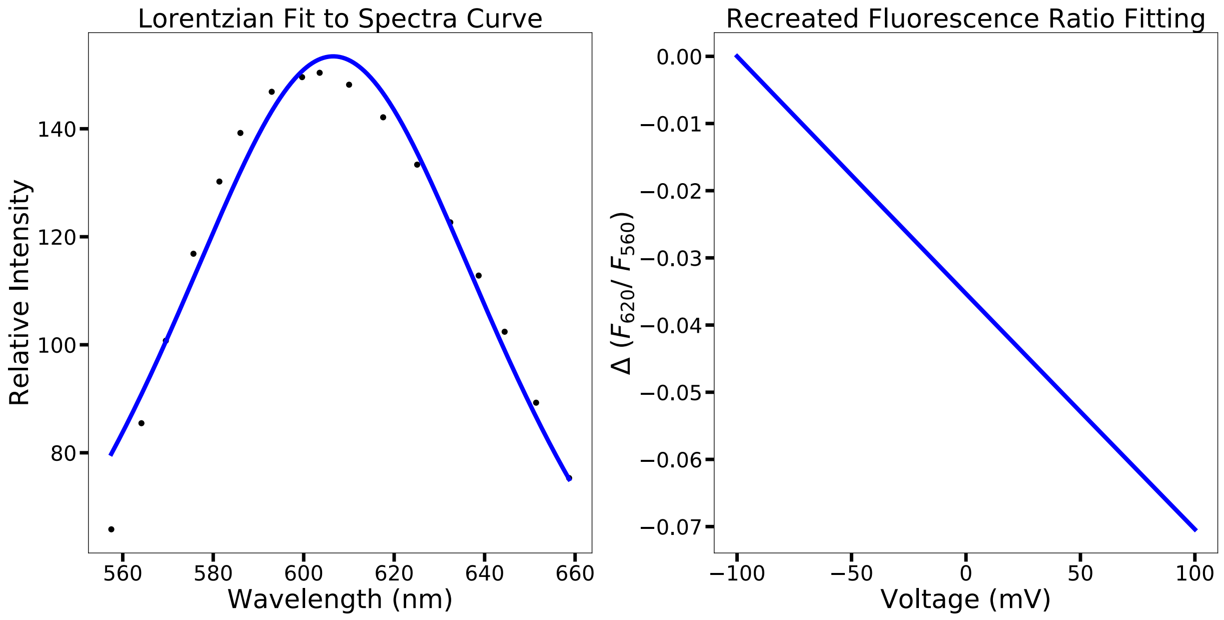

Figure S9: Recreation of Kao et al's data[S12] to allow for direct comparison - on the left is the fitting of their emission peak at -80 mV with a Lorentzian function $153.4\left[1+\left((\lambda-\lambda_{\max})/51.1\right)^2\right]^{-1}$. On the right is plotting the change in the ratio of the fluorescence intensity at wavelengths of 620 nm and 560 nm using that Lorentzian function: $\Delta(F_{620}/F_{560}) = F(620;V_{\mathrm{mp}})/F(560;V_{\mathrm{mp}}) - F(620;-100\mathrm{mV})/F(560;-100\mathrm{mV})$ to match the experimentally determined linear relationship $-0.000352(x) - 0.0352$.

References

(S1) Huang, L.; Roux, B. *Journal of chemical theory and computation* **2013**, *9*, 3543–3556.

(S2) others,, et al. *Journal of computational chemistry* **2009**, *30*, 1545–1614.

(S3) Robinson, D.; Besley, N. A.; OShea, P.; Hirst, J. D. *J. Phys. Chem. B* **2011**, *115*, 4160–4167.

(S4) Rusu, C. F.; Lanig, H.; Othersen, O. G.; Kryschi, C.; Clark, T. *J. Phys. Chem. B* **2008**, *112*, 2445–2455.

(S5) Neese, F. *Wiley Interdisciplinary Reviews: Computational Molecular Science* **2012**, *2*, 73–78.

(S6) Neese, F. *Wiley Interdisciplinary Reviews-Computational Molecular Science* **2017**, *8*, 73–78.

(S7) Gross, D.; Loew, L. M.; Webb, W. W. *Biophysical journal* **1986**, *50*, 339–348.

(S8) Lojewska, Z.; Farkas, D.; Ehrenberg, B.; Loew, L. *Biophysical journal* **1989**, *56*, 121–128.

(S9) Ehrenberg, B.; Farkas, D. L.; Fluhler, E. N.; Lojewska, Z.; Loew, L. M. *Biophysical journal* **1987**, *51*, 833–837.

(S10) Müller, W.; Windisch, H.; Tritthart, H. *Biophysical journal* **1989**, *56*, 623–629.

(S11) Chien, C.-B.; Pine, J. *Biophysical journal* **1991**, *60*, 697–711.

(S12) Kao, W.; Davis, C. E.; Kim, Y.; Beach, J. *Biophysical journal* **2001**, *81*, 1163–1170.
