# Supplementary material for: Simulating the Voltage-Dependent Fluorescence of Di-8-ANEPPS in a Lipid Membrane
Source: J Phys Chem Lett. 2023 Sep 7;14(36):8268–76. doi: 10.1021/acs.jpclett.3c01257 (PMC10510438; doi:10.1021/acs.jpclett.3c01257)
Supplement: Supplementary file 2 — jz3c01257_si_002.pdf [file jz3c01257_si_002.pdf]

jz-2023-01257x.R1

Name: Peer Review Information for "Simulating the Voltage-Dependent Fluorescence of Di-8-ANEPPS in a Lipid Membrane"

First Round of Reviewer Comments

Reviewer: 1

Comments to the Author

jz-2023-01257x employs theoretical chemistry methods to simulate the voltage-dependent fluorescence of Di-8-ANEPPS in a lipid membrane, proposing also comparisons with experiment. The work is potentially of general interest but in its current form it presents severe drawbacks.

1) the theoretical chemistry methodology is based on Ref. 23, which is not available. As a matter of fact, the information on the method are not sufficient to allow reproduction of the data and analysis. Similarly, the reader has no clue on the reliability of the employed methods, their strengths and limitations.

2) what is the accuracy of the re-parameterized FF to reproduce ab initio structures, for both the ground and dominant excited state? This should be stated somewhere (typically SI) to be self-contained.

3) which method was employed to obtain the optical properties of embedded di-8-ANEPPS? Even if the methodology was discussed in length in the not-available Ref. 23, it appears necessary to say a few words here as well.

4) the results are based on MD results. Averages have been calculated. How many snapshots were used for computing these averages? For many results the standard deviations (or related information) are not provided, while they are necessary to assess the associated analysis.

5) a potential/voltage/electric field has been applied on the dyed membrane and the dynamics was run in this situation for a given period of time. Was this amount of time sufficient to reach equilibrium? This major issue does not seem to be addressed.

6) a 37.8° value is given in page 9 . Is it related to the GS or to the ES?

7) the orientation of the dye is discussed by considering the N-N axis. This does not appear to be the most relevant in presence of an external electric field. Naturally, it would have appeared better to provide and analyze the angle between the field and the electric dipole moment (because it is usually known to orient along the external field).

8) what is the rationale of using Lorentzian functions in the wavelength space? Shouldn't it be excitation energies ? (or wavenumber).

Reviewer: 2

Comments to the Author

In this work by Youngworth and Roux, classical MD simulations of di-8-ANEPPS are presented to simulate the voltage-dependent fluorescence within a DPPC bilayer. This work represents a significant advance in the application of classical MD to simulate chromophores / fluorophores in a voltage sensitive environment. As such, this work is publishable in the Journal of Physical Chemistry Letters. I recommend a couple of very minor points to the authors:

1) In the abstract / introduction, be clear whether the “membrane potential” is an external applied potential and/or the membrane dipole potential.

2) Figure 1 – a zoomed-in region showing 5-20 Å to demonstrate more clearly which part(s) of di-8-ANEPPS are in the gradient region of the potential would be useful for the reader.

3) page 15 (figure 5). In the main text discussing this figure, please clarify why the region of -100 mV to +100 mV is chosen for the straight-line fit.

Author's Response to Peer Review Comments:

Dear Professor Editor,

We have revised the manuscript according to the comments of the two Reviewers and the instructions from the journal. A point-by-point response is included. For your convenience, PDF files of the manuscript and the supplementary information with all changes marked in blue have been uploaded. A clean unmarked PDF file of the supplementary information has also been uploaded for publication.

As additional information for the Reviewers, we have also uploaded the PDF of the manuscript "Simulating the Fluorescence of Di-8-ANEPPS in Solvents of Different Polarity" that is currently under review at the Journal of Physical Chemistry.

We hope that the revised manuscript will be suitable for publication. We sincerely thank you for your attention to our manuscript.

Best regards,

Benoit Roux

Response to Reviewer 1

Recommendation: This paper may be publishable, but major revision is needed; I would like to be invited to review any future revision.

Comments: jz-2023-01257x employs theoretical chemistry methods to simulate the voltage-dependent fluorescence of Di-8-ANEPPS in a lipid membrane, proposing also comparisons with experiment. The work is potentially of general interest but in its current form it presents severe drawbacks.

1) the theoretical chemistry methodology is based on Ref. 23, which is not available. As a matter of fact, the information on the method are not sufficient to allow reproduction of the data and analysis. Similarly, the reader has no clue on the reliability of the employed methods, their strengths and limitations.

As mentioned in the text, the ground and excited states were parameterized on the basis of CASSCF calculations at the def2-SVP def2-SVP/C level with orbstep SuperCi and switchstep DIIS convergence criteria included that were carried out using the program ORCA. We realize that the manuscript implicitly relies on additional information from reference 23 (currently under review), which is not immediately available to the Reviewer..We now provide the whole of reference 23 as additional information (not to be published). Furthermore, we also include most of the computational methodology in Supplementary Information and revised the manuscript to provide more of the key details about the methodology for the sake of completeness.

2) what is the accuracy of the re-parameterized FF to reproduce ab initio structures, for both the ground and dominant excited state? This should be stated somewhere (typically SI) to be self-contained.

The optimal geometries are well reproduced by the force field. As the main objective is to characterize the influence of the environment, the most important aspect of the force field concerns the fixed electrostatic charges for the ground and excited states. The fixed electrostatic charges were optimized to reproduce the CASSCF(6,6) calculations for the di-4-ANEPPS molecule, yielding a dipole of 31.23 Debye and 41.40 Debye for the ground and excited state, which is a change of 10.17 Debye. The CASSCF(6,6) calculation of di-4-ANEPPS yielded a dipole of 22.34 and 33.14 Debye for the ground and excited state, respectively, with a very similar change of 10.80 Debye. This is also consistent with a previous QM study reporting a ground state dipole of 36.7$\pm$2.7 Debye and an excited state dipole of 48.3$\pm$2.6 Debye, for a total change of 11.4$\pm$1.7 Debye (Rusu et al, 2008). The force field does not account for induced polarization.

3) which method was employed to obtain the optical properties of embedded di-8-ANEPPS? Even if the methodology was discussed in length in the not-available Ref. 23, it appears necessary to say a few words here as well.

CASSCF(6,6) calculations on the di-4-ANEPPS molecule were used to characterize di-8-ANEPPS. The parameters of the longer hydrocarbon chain is simply taken from the standard CHARMM force field.

4) the results are based on MD results. Averages have been calculated. How many snapshots were used for computing these averages? For many results the standard deviations (or related information) are not provided, while they are necessary to assess the associated analysis.

All MD simulations were 1 microsecond long. The configurations along the trajectory at 0 mV were every 100 ps, yielding a total of 10,000 snapshots. But the simulations at larger voltages were saved more frequently and have 100,000 snapshots. As requested, we have estimated the standard uncertainty on the averages calculated from MD for both the absorption and emission wavelenths (in nm) using block averages:

```
-500 mV   464.8507  +/-  0.074
-100 mV   460.4471  +/-  0.069
   0 mV   460.5651  +/-  0.272
 100 mV   459.3372  +/-  0.069
 500 mV   457.4763  +/-  0.073

emission
-500 mV   608.5598  +/-  0.141
-100 mV   606.5503  +/-  0.143
   0 mV   605.5221  +/-  0.535
 100 mV   605.1193  +/-  0.138
 500 mV   604.0569  +/-  0.141
```

Interestingly, the statistical uncertainty is slightly larger for the emission, presumably because there are more fluctuations in the membrane-anchoring of the di-8-ANEPPS molecule in its excited state compared to the ground state.

5) a potential/voltage/electric field has been applied on the dyed membrane and the dynamics was run in this situation for a given period of time. Was this amount of time sufficient to reach equilibrium? This major issue does not seem to be addressed.

All the MD simulations with and without applied electric field were 1 microsecond long. Previous studies of membrane potential have shown that the average electrostatic profile settles very rapidly, within about 10-50 nanoseconds (Gumbart et al. 2012 Feb;1818(2):294-302. doi: 10.1016/j.bbamem.2011.09.030.) The text was revised for clarification.

6) a 37.8° value is given in page 9 . Is it related to the GS or to the ES?

This statement is from an experimental paper by Lambacher et al (2001) who reported the orientation of di-8-ANEPPS in lipid membrane measured by fluorescence interferometry on a silicon chip. They stated that this is the angle of the transition dipole moment with respect to the membrane normal. The text was clarified.

7) the orientation of the dye is discussed by considering the N-N axis. This does not appear to be the most relevant in presence of an external electric field. Naturally, it would have appeared better to provide and analyze the angle between the field and the electric dipole moment (because it is usually known to orient along the external field).

On average, the excited state dipole makes an angle of about 12° with the N-N vector during the simulations with di-8-ANEPPS in its excited state.

Furthermore, we determined the tilt angle between the dipole and the membrane normal. For the simulations that used the ground state parameters we used the ground state dipole, and for simulation that used the excited state parameters we used the excited state dipole. Shown are the averages with standard deviation. Here are the angles (in degrees):

```
Ground state
-500 mV   27.03  +/- 14.2
-100 mV   25.96  +/- 13.4
   0 mV   27.13  +/- 14.3
```

```
 100 mV   27.69  +/- 14.4
 500 mV   29.23  +/- 15.2

Excited state
-500 mV   45.12  +/- 16.5
-100 mV   47.52  +/- 16.2
   0 mV   46.01  +/- 17.4
 100 mV   46.95  +/- 15.9
 500 mV   47.01  +/- 15.8
```

These numbers are really close to the N-N vector angle to the membrane normal, which for the ground state increased from 26.2 to 28.2 (27.0 to 29.2 for the dipole angle) and for the excited state increased from 47.9 to 49.5 (45.1 to 47.0 for the dipole angle).

Ultimately, any axis to determine one angle will rely on a somewhat subjective choice. For most biophysicists it is helpful to have a simpler structural determination of the able based on the position of the atoms. Indeed the electric dipole can be a reasonable vector to use, although it is not without some issues. For instance, the dipole of the entire molecule is not entirely relevant because some parts that carry a large charge such as the sulphonate group are not located within the transmembrane potential field. This implies that only the dipole within the transmembrane region is truly relevant. This point was clarified in the manuscript. .

8) what is the rationale of using Lorentzian functions in the wavelength space? Shouldn't it be excitation energies ? (or wavenumber).

The assumed Lorentzian lineshape was fitted to the published experimental data. This functional form, which matches well the published data, was mainly used to convert the reported ratio emission at two wavelengths into a shift of the maximum wavelength (which was not reported). Other similar lineshape functions might have been used as long as they reproduce the experimental data.

Response to Reviewer 2

Recommendation: This paper is publishable subject to minor revisions noted. Further review is not needed.

Comments:

In this work by Youngworth and Roux, classical MD simulations of di-8-ANEPPS are presented to simulate the voltage-dependent fluorescence within a DPPC bilayer. This work represents a significant advance in the application of classical MD to simulate chromophores / fluorophores in a voltage sensitive environment. As such, this work is publishable in the Journal of Physical Chemistry Letters. I recommend a couple of very minor points to the authors: 1) In the abstract / introduction, be clear whether the "membrane potential" is an external applied potential and/or the membrane dipole potential.

2) Figure 1 – a zoomed-in region showing 5-20 Å to demonstrate more clearly which part(s) of di-8-ANEPPS are in the gradient region of the potential would be useful for the reader.

Good suggestion. Figure 1 was revised accordingly.

3) page 15 (figure 5). In the main text discussing this figure, please clarify why the region of -100 mV to +100 mV is chosen for the straight-line fit.

We initially ran some simulations at ±500 mV because we were worried and wanted to make sure of generating a sufficiently big signal to test the calculations. We still include this result in the figure, but most experimental measurements are carried out with voltages that do not exceed ±100 mV. Unsurprisingly, at higher voltages there seems to be some non-linearity in the response of the dye, presumably because its orientation and position begins to be affected by thevoltage.

Formatting Notes from the Editorial Office

1. Using acronyms in the title is discouraged. Please spell out all acronyms in the title of the manuscript and Supporting Information.

ACS journals have typically accepted the word "di-8-ANEPPS" in the title

Charlotte Bouquiaux, Frédéric Castet, Benoît Champagne. Unravelling the Effects of Cholesterol on the Second-Order Nonlinear Optical Responses of Di-8-ANEPPS Dye Embedded in Phosphatidylcholine Lipid Bilayers. J Phys Chem B. 2021 Sep 16;125(36):10195-10212.

Charlotte Bouquiaux, Frédéric Castet, Benoît Champagne. Influence of the Nature of the Lipid Building Blocks on the Second-Order Nonlinear Optical Responses of an Embedded Di-8-ANEPPS Probe. J Phys Chem B. 2023 Jan 19;127(2):528-541.

David Robinson, Nicholas A Besley, Paul O'Shea, Jonathan D Hirst. Di-8-ANEPPS emission spectra in phospholipid/cholesterol membranes: a theoretical study. J Phys Chem B. 2011 Apr 14;115(14):4160-7.

2. Please add the city, state, and postal code to the second affiliation

Done.

3. Please shorten the abstract to 150 words or fewer.

The revised abstract has 149 words.

4. Remove the section heading(s) throughout the body of the manuscript (you can leave Methods and Abstract headings).

Done.

5. One or more of your figures and tables includes a reference notation. Please confirm that this pertains only to data and not the figure itself. If it pertains to the use of a published image, permissions must be secured for any graphics NOT originally published by ACS or for Open Access content. Permission is needed if you are using another publisher's or copyright owner's figures/tables verbatim, adapting/modifying them, or using them in part. If the images are from an Open Access publisher, please confirm.

All figures are original. The references only means that some datapoints were extracted from previous publications. This was clarified in the text.

6. A brief, nonsentence description of the actual contents of each supporting information file is required. This description should be labeled Supporting Information and should appear before the Acknowledgement and Reference sections. Examples of sufficient and insufficient descriptions are as follows: *Examples of sufficient descriptions: "Supporting Information: 1H NMR spectra for all compounds" or "Additional experimental details, materials, and methods, including photographs of experimental setup". *Examples of insufficient descriptions: "Supporting Information: Figures S1-S3" or "Additional figures as mentioned in the text".

Done.

7. In both the main file and the supporting information, fix the style of all references to use JPCL formatting (check all references carefully). ***JPC Letters reference formatting requires that journal references should contain: () around numbers, author names, article title (titles entirely in title case or entirely in lower case), abbreviated journal title (italicized), year (bolded), volume (italicized), and pages (first-last). Book references should contain author names, book title (in the same pattern), publisher, city, and year. Websites must include date of access.

Done.

8. Please add full publication information to reference 23 and 33.

This manuscript is under review at the Journal of Physical Chemistry. We may have a complete reference soon.

9. There is a discrepancy between the title listed on the supporting information file vs. the title on the online submission form/manuscript file. Please correct the title so that it is the same in all locations.

Done.

10. Please add the author affiliations to the title page of the SI file.

Done.

11. Please include annotated version(s) of your revised publication file(s) with colored text or highlights indicating the revisions that you have made, and upload them as “Supporting Information for Review Only." Please also upload “clean” copies for publication. (No highlighting, annotations, or colored text.)

Done.

jz-2023-01257x.R2

Name: Peer Review Information for "Simulating the Voltage-Dependent Fluorescence of Di-8-ANEPPS in a Lipid Membrane"

Second Round of Reviewer Comments

Reviewer: 1

Comments to the Author

The authors have addressed successfully all the comments raised by the reviewers (a lot of the answers are given in the now-shared Ref. 23, prior publication). The paper is therefore recommended for publication.

Note that, in the SI, there are a few "??" that should be adapted to the actual Figure numbers.

Author's Response to Peer Review Comments:

We have complied with all the requests:

1. Please add an Abstract heading above the abstract.

Added "Abstract" heading.

2. A brief, nonsentence description of the actual contents of each supporting information file is required in the manuscript file. This description should be labeled Supporting Information and should appear before the Acknowledgement and Reference sections.

Added this just above Acknowledgements:

Supporting Information

Supporting Information: the force field parameterization of di-8-ANEPPS, membrane potential profiles, tracked positioning of key atoms of di-8-ANEPPS over the course of simulation, and details on how experimental data was handled for direct comparison.

3. For references 23 and 33, please add "in review" or publication information if available

For reference 23:

Youngworth, R.; Roux, B. Simulating the Fluorescence of Di-8-ANEPPS in Solvents of Different Polarity. J Phys Chem B 2023 (in review).

For reference 33: Wu, E. L.; Cheng, X.; Jo, S.; Rui, H.; Song, K. C.; Dávila-Contreras, E. M.; Qi, Y.; Lee, J.; Monje-Galvan, V.; Venable, R. M. et al. CHARMM-GUI Membrane Builder toward realistic biological membrane simulations. J Comput Chem 2014, 35, 1997– 2004.

4. In the SI file, please replace all question marks in the "Supplemental Figure ??" citations.

One figure S1 was incorrectly assigned. This was fixed now. There are no ?? in the SI.
